# Scene Construction, Visual Foraging, and Active Inference

**DOI:** 10.3389/fncom.2016.00056

**Published:** 2016-06-14

**Authors:** M. Berk Mirza, Rick A. Adams, Christoph D. Mathys, Karl J. Friston

**Affiliations:** ^1^Wellcome Trust Centre for Neuroimaging, Institute of Neurology, University College LondonLondon, UK; ^2^Division of Psychiatry, University College LondonLondon, UK; ^3^Institute of Cognitive Neuroscience, University College LondonLondon, UK; ^4^Translational Neuromodeling Unit, Institute for Biomedical Engineering, University of Zurich and Swiss Federal Institute of Technology in Zurich (ETHZ)Zurich, Switzerland; ^5^Max Planck UCL Centre for Computational Psychiatry and Ageing ResearchLondon, UK

**Keywords:** active inference, visual search, Bayesian inference, scene construction, free energy, information gain, epistemic value, salience

## Abstract

This paper describes an active inference scheme for visual searches and the perceptual synthesis entailed by scene construction. Active inference assumes that perception and action minimize variational free energy, where actions are selected to minimize the free energy expected in the future. This assumption generalizes risk-sensitive control and expected utility theory to include epistemic value; namely, the value (or salience) of information inherent in resolving uncertainty about the causes of ambiguous cues or outcomes. Here, we apply active inference to saccadic searches of a visual scene. We consider the (difficult) problem of categorizing a scene, based on the spatial relationship among visual objects where, crucially, visual cues are sampled myopically through a sequence of saccadic eye movements. This means that evidence for competing hypotheses about the scene has to be accumulated sequentially, calling upon both prediction (planning) and postdiction (memory). Our aim is to highlight some simple but fundamental aspects of the requisite functional anatomy; namely, the link between approximate Bayesian inference under mean field assumptions and functional segregation in the visual cortex. This link rests upon the (neurobiologically plausible) process theory that accompanies the normative formulation of active inference for Markov decision processes. In future work, we hope to use this scheme to model empirical saccadic searches and identify the prior beliefs that underwrite intersubject variability in the way people forage for information in visual scenes (e.g., in schizophrenia).

## Introduction

We have a remarkable capacity to sample our visual world in an efficient fashion, resolving uncertainty about the causes of our sensations so that we can act accordingly. This capacity calls on the ability to optimize not just beliefs about the world that is “out there” but also the way in which we sample information (Howard, 1966; Shen et al., 2011; Wurtz et al., 2011; Andreopoulos and Tsotsos, 2013; Pezzulo et al., 2013). This is particularly evident in active vision, where discrete and restricted (foveal) visual data is solicited every few 100 ms, through saccadic eye movements (Grossberg et al., 1997; Srihasam et al., 2009). In this paper, we consider the principles that underlie this visual foraging—and how it is underwritten by resolving uncertainty about the visual scene that is being explored. We approach this problem from the point of view of active inference; namely, the assumption that action and perception serve to minimize surprise or uncertainty under prior beliefs about how sensations are caused.

Active or embodied inference is a corollary of the free energy principle that tries to explain everything in terms of the minimization of variational free energy. Variational free energy is a proxy for surprise or Bayesian model evidence. This means that minimizing free energy corresponds to avoiding surprises or maximizing model evidence. The embodied or situated aspect of active inference acknowledges the fact that we are the authors of the sensory evidence we garner. This means that the consequences of sampling or action must themselves be inferred. In turn, this implies that we have (prior) beliefs about our behavior. Active inference assumes that the only self-consistent prior belief is that our actions will minimize free energy; in other words, we (believe we) will behave to avoid surprises or resolve uncertainty through active sampling of the world. This paper illustrates how this works with simulations of saccadic eye movements and scene construction using a discrete (Markov decision process) formulation of active inference (Friston et al., 2011, 2015).

We consider the problem of categorizing a scene based upon the sequential sampling of local visual cues to construct a picture or hypothesis about how visual input is generated. This is essentially the problem of scene construction (Hassabis and Maguire, 2007; Zeidman et al., 2015), where each scene corresponds to a hypothesis or explanation for sequences of visual cues. The main point that emerges from this perspective is that the scene exists only in the eye of the beholder: it is represented in a distributed fashion through recurrent message passing or belief propagation among functionally segregated representations of where (we are currently sampling) and what (is sampled). This application of active inference emphasizes the epistemic value of free energy minimizing behavior—as opposed to the pragmatic (utilitarian) value of searching for preferred outcomes. However, having said this, the theory (resp. simulations) uses exactly the same mathematics (resp. software routines) that we have previously used to illustrate foraging behavior in the context of reward seeking (Friston et al., 2015).

Our aim is to introduce a model of epistemic foraging that can be applied to empirical saccadic eye movements and, ultimately, be used to phenotype individual subjects in terms of their prior beliefs: namely, the prior precision of beliefs about competing epistemic policies and the precision of prior preferences (c.f., “incentive epistemic” and motivational salience). This may be particularly interesting when looking at schizophrenia and other clinical phenotypes that show characteristic abnormalities during visual (saccadic) exploration. For example, schizophrenia has been associated with “aberrant salience,” in which subjects attend to—and hence saccade to—inconsequential features of the environment (Kapur, 2003; Beedie et al., 2011). It is unclear, however, whether “aberrant” salience is epistemic or motivational, or both; put simply, do subjects with schizophrenia fail to gather information, and/or fulfill their goals?

This paper comprises three sections. In the first, we briefly rehearse active inference and the underlying formalism. The second section describes the paradigm that it subsequently modeled using the formalism of the first section. In brief, this requires agents to categorize a scene based upon discrete (visual) cues that can be sampled from one of four peripheral locations (starting from central fixation). Crucially, the scene category is determined purely by the spatial relationships among the cues—as opposed to their absolute position. By equipping the agent's generative model with preferences for correct (as opposed to incorrect) feedback, we also model the overt reporting of categorical decisions; thereby emulating a speeded response task. In the final section, we characterize sequences of trials under different levels of prior precision and preferences (for avoiding incorrect feedback). The results are characterized in terms of simulated electrophysiological responses, saccadic intervals, and the usual behavioral measures of speed and accuracy. We conclude with a brief discussion of how this model might be used in an empirical (computational psychiatry) setting.

## Active inference and epistemic value

Active inference is based upon the premise that every living thing minimizes variational free energy. This single premise leads to some surprisingly simple update rules for action, perception, policy selection, and the encoding of salience or precision. In principle, the active inference scheme described below can be applied to any paradigm or choice behavior. Indeed, earlier versions have already been used to model waiting games (Friston et al., 2013), two-step maze tasks (Friston et al., 2015), the urn task and evidence accumulation (FitzGerald et al., 2015), trust games from behavioral economics (Moutoussis et al., 2014), addictive behavior (Schwartenbeck et al., 2015) and engineering benchmarks such as the mountain car problem. It has also been used in the setting of computational fMRI (Schwartenbeck et al., 2014).

### Active inference and generative models

Active inference rests upon a generative model of observed outcomes. This model is used to infer the most likely causes of outcomes in terms of expectations about states of the world. These states are called *hidden states* because they can only be inferred indirectly through, possibly limited, sensory observations. Crucially, observations depend upon action, which requires the generative model to entertain expectations under different policies or action sequences. Because the model generates the consequences of sequential action, it has explicit representations of the past and future; in other words, it is equipped with a working memory and expectations about future (counterfactual) states of the world under competing policies. These expectations are optimized by minimizing variational free energy, which renders them (approximately) the most likely (posterior) expectations about states of the world, given the current observations.

Expectations or beliefs about the most likely policy are based upon the prior belief that policies are more likely if they pursue a trajectory or path that has the least free energy (or greatest model evidence). As we will see below, this expected free energy can be expressed in terms of epistemic and extrinsic value, where epistemic value scores the information gain or reduction in uncertainty about states of the world—and extrinsic value depends upon prior beliefs about future outcomes. These prior preferences play the role of utility in economics and reinforcement learning.

Having evaluated the relative probability of different policies, expectations under each policy can then be averaged in proportion to their (posterior) probability. In statistics, this is known as *Bayesian model averaging*. The results of this averaging specify the next most likely outcome, which determines the next action. Once an action has been selected, it generates a new outcome and the (perception and action) cycle starts again. The resulting behavior is a principled interrogation and sampling of sensory cues that has both epistemic and pragmatic aspects. Generally, behavior in an ambiguous and uncertain context is dominated by epistemic drives until there is no further uncertainty to resolve—and extrinsic value predominates. At this point, explorative behavior gives way to exploitative behavior. In this paper, we are primarily interested in the epistemic behavior, and only use extrinsic value to encourage the agent to report its decision, when it is sufficiently confident.

**Figure 1:**
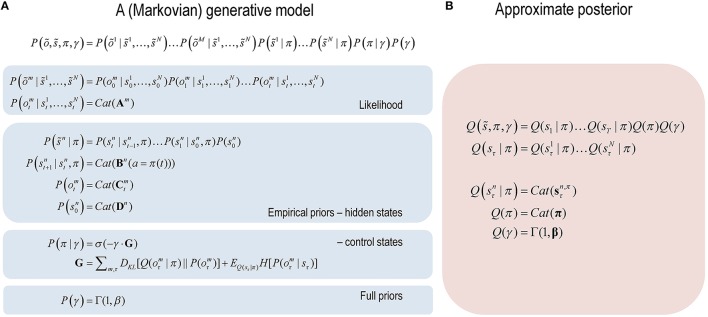
**Formal specification of the generative model and (approximate) posterior. (A)** These equations specify the form of the (Markovian) generative model used in this paper. A generative model is essentially a specification of the joint probability of outcomes or consequences and their (latent or hidden) causes. Usually, this model is expressed in terms of a *likelihood* (the probability of consequences given causes) and priors over the causes. When a prior depends upon a random variable it is called an *empirical prior*. Here, the generative model specifies the mapping between hidden states and observable outcomes in terms of the likelihood. The priors in this instance pertain to transitions among hidden states that depend upon action, where actions are determined probabilistically in terms of policies (sequences of actions). The key aspect of this generative model is that, *a priori*, policies are more probable if they minimize the (path integral of) expected free energy **G**. Bayesian model inversion refers to the inverse mapping from consequences to causes; i.e., estimating the hidden states and other variables that cause outcomes. **(B)** In variational Bayesian inversion, one has to specify the form of an approximate posterior distribution, which is provided on the right panel. This particular form uses a mean field approximation, in which posterior beliefs are approximated by the product of marginals or factors. Here, a mean field approximation is applied both to posterior beliefs at different points in time and different sorts of hidden states. See the main text and Table 1 for more detailed explanation of the variables.

In more detail: expectations about hidden states (and the precision of beliefs about competing policies) are updated to minimize variational free energy under a generative model. The generative model considered here is fairly generic (see Figure 1). Outcomes at any particular time depend upon hidden states, while hidden states evolve in a way that depends upon action. Formally, this model is specified by two sets of matrices (strictly speaking these are multidimensional arrays). The first, A^m^, maps from hidden states to the *m*-th outcome and can embody ambiguity in the outcomes generated by any particular state. The *m*-th sort of outcome here can be considered the *m*-th modality; for example, exteroceptive or proprioceptive observations. The second set of matrices B^n^(*a*), prescribe the transitions among the *n*-th hidden states, under an action, *a*. The *n*-th sort of hidden state can correspond to different factors or attributes of the world; for example, the location of an object and its identity. The remaining parameters encode prior beliefs about the initial states D^n^, the precision of beliefs about policies γ = 1/β, where a policy returns an action at a particular time *a* = π(*t*) and prior preferences C^m^ that define the expected free energy (see below).

**Figure 2:**
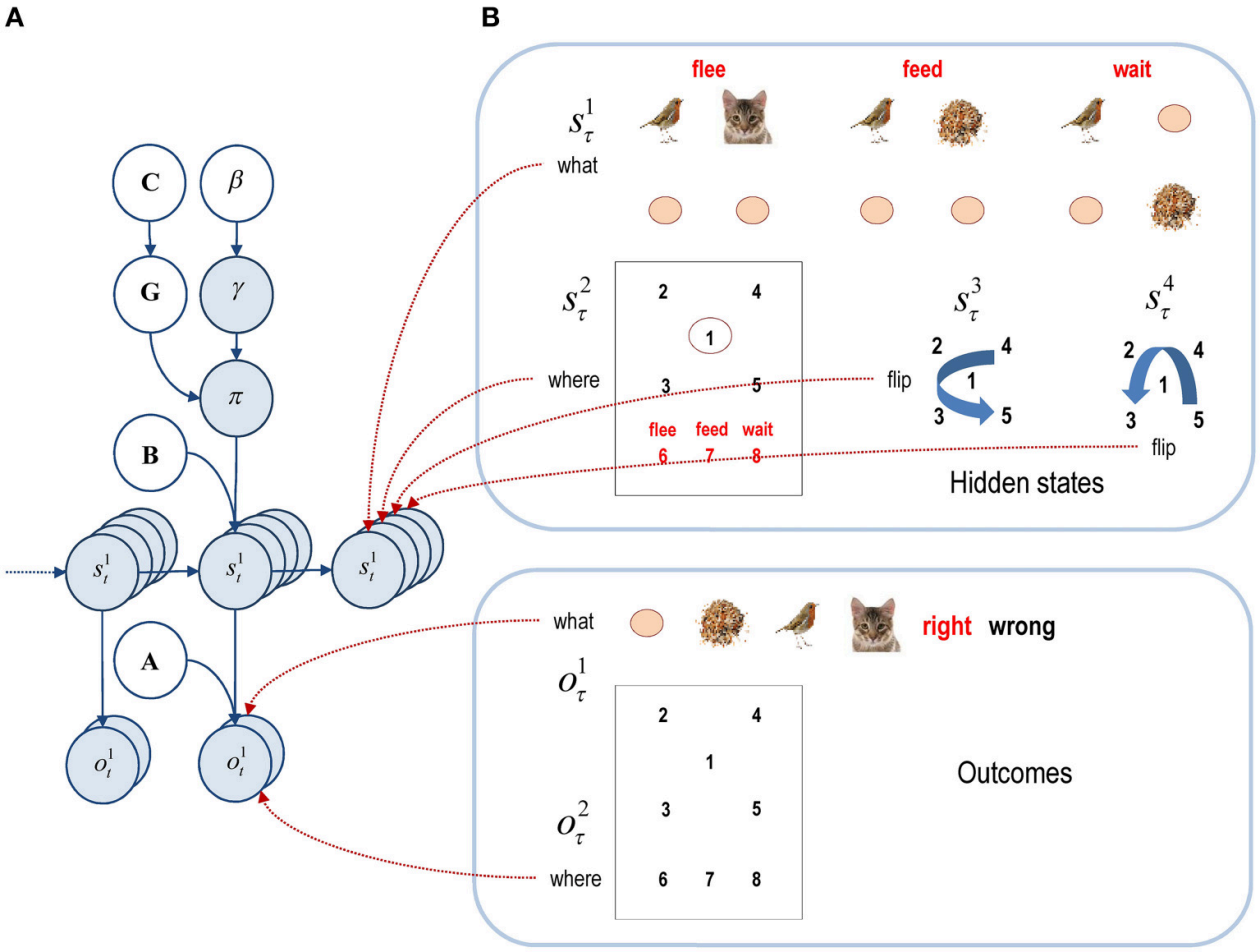
**Graphical model corresponding to the generative model. (A)** The left panel shows the conditional dependencies implied by the generative model of previous figure. Here, the variables in white circles constitute (hyper) priors, while the blue circles denote random variables. This format shows how outcomes are generated from hidden states that evolve according to probabilistic transitions, which depend on policies. The probability of a particular policy being selected depends upon expected free energy and a precision or inverse temperature. **(B)** The right panels show an example of different hidden states and outcomes modalities. This particular example will be used later to model perceptual categorization in terms of three scenarios or scenes (*flee, feed*, or *wait*). The two outcome modalities effectively report what is seen and where it is seen. See the main text for a more detailed explanation.

The form of the generative model in Figure 1 means that outcomes are generated in the following way: first, a policy is selected using a softmax function of expected free energy for each policy, where the inverse temperature or precision is selected from a prior (exponential) density. Sequences of hidden states are then generated using the probability transitions specified by a selected policy. These hidden states then generate outcomes in several modalities. Figure 2 (left panel) provides a graphical summary of the dependencies implied by the generative model in Figure 1. Perception or inference corresponds to inverting or fitting this generative model, given a sequence of outcomes. This corresponds to optimizing the expected hidden states, policies and precision with respect to variational free energy. These (posterior) estimates constitute posterior beliefs, usually denoted by the probability distribution *Q*(*x*), where $x=\tilde{s},\pi ,\gamma$ are the hidden or unknown variables.

### Variational free energy and inference

In variational Bayesian inference, model inversion entails minimizing variational free energy with respect to the sufficient statistics (i.e., parameters) of the posterior beliefs (see Figure 1, right panel and Table 1 for a glossary of expressions):

(1) $$\begin{array}{l} \begin{array}{l} Q\left(x\right)=\text{arg}{\text{min}}_{Q\left(x\right)}F\\ \approx P\left(x|õ\right)\\ F=E_{Q}\left[\text{ln }Q\left(x\right)-\text{ln }P\left(õ|x\right)-\text{ln }P\left(x\right)\right]\\ =E_{Q}\left[\text{ln }Q\left(x\right)-\text{ln }P\left(x|õ\right)-\text{ln }P\left(õ\right)\right]\\ =\underset{relativeentropy}{\underset{︸}{D\left[Q\left(x\right)||P\left(x|õ\right)\right]}}-\underset{logevidence}{\underset{︸}{\text{ln }P\left(õ\right)}}\\ =\underset{complexity}{\underset{︸}{D\left[Q\left(x\right)||P\left(x\right)\right]}}-\underset{accuracy}{\underset{︸}{E_{Q}\left[\text{ln }P\left(õ|x\right)\right]}} \end{array} \end{array}$$

where õ = (*o*_1_, …, *o*_*t*_) denotes observations up until the current time.

**Table 1:** **Glossary of expressions**.

**Expression**	**Description**
$o_{\tau }=(o_{\tau }^{1},\ldots ,o_{\tau }^{M})\in \{0,1\},o_{\tau }\in [0,1],{\overset{⌢}{o}}_{\tau }=\ln o_{\tau }$	Outcomes in *M* modalities, their posterior expectations and logarithms
õ = (*o*_1_, …, *o*_*t*_)	Sequences of outcomes up until the current time
$s_{\tau }=(s_{\tau }^{1},\ldots ,s_{\tau }^{N})\in \{0,1\},s_{\tau }\in [0,1],{\overset{⌢}{s}}_{\tau }=\ln s_{\tau }$	Hidden states in *N* factors, their posterior expectations and logarithms
$\tilde{s}=\left(s_{1},\ldots ,s_{T}\right)$	Sequences of hidden states up until the end of the current trial
$\pi =(\pi_{1},\ldots ,\pi_{K}),\;\pi \in [0,1],\overset{⌢}{\pi }=\ln\pi$	Policies specifying action sequences, their posterior expectations and logarithms
*a* = π(*t*) = (*a*_1_, …, *a*_*N*_)	Action or control variables for each factor of hidden states
$\gamma ,\;\gamma =\frac{1}{\beta }$	The precision (inverse temperature) of beliefs about policies and its posterior expectation
β	Prior expectation of temperature (inverse precision) of beliefs about policies
**A**^*m*^	Likelihood array mapping from hidden states to the *m*-th modality
${\text{B}}^{n}\left(a\right),{\text{B}}_{\tau }^{n,\pi }={\text{B}}^{n}\left(a=\pi \left(\tau \right)\right)$	Transition probability for the *n*-th hidden state under each action
${\text{C}}_{\tau }^{m}$	Logarithm of the prior probability of the *m*-th outcome; i.e., preferences or utility
**D**^*n*^	Prior expectation of the *n*-th hidden state at the beginning of each trial
**F:F**${}_{\pi }=F\left(\pi \right)=\underset{\tau }{\sum }F\left(\pi ,\tau \right)=\underset{n,\tau }{\sum }F\left(\pi ,\tau ,n\right)$	Variational free energy for each policy
**G:G**${}_{\pi }=G\left(\pi \right)=\underset{\tau }{\sum }G\left(\pi ,\tau \right)=\underset{m,\tau }{\sum }G\left(\pi ,\tau ,m\right)$	Expected free energy for each policy
**H**^m^	Entropy of the *m*-th outcome
**A**· **s**${}^{∙}=\underset{i,j,k\ldots }{\sum }{\text{A}}_{i,j,k\ldots }{\text{s}}_{i}^{1}{\text{s}}_{j}^{2}{\text{s}}_{k}^{3}\ldots$	Dot product (or sum of products), returning a scalar
**A**· **s**^∕*n*^	A dot product over all but the *n*-th vector

#### Remarks

Because the relative entropy (or Kullback-Leibler divergence) cannot be less than zero, the penultimate equality means that free energy is minimized when the approximate posterior becomes the true posterior. At this point, the free energy becomes the negative log evidence for the generative model (Beal, 2003). This means minimizing free energy is equivalent to maximizing model evidence, which is equivalent to minimizing the complexity of accurate explanations for observed outcomes (last equality above).

Minimizing free energy ensures expectations encode posterior beliefs, given observed outcomes. However, beliefs about policies rest on future outcomes. This means that policies should, *a priori*, minimize the free energy of beliefs about the future. This can be formalized by making the log probability of a policy proportional to the free energy expected in the future (Friston et al., 2015):

$$\begin{array}{l} G\left(\pi \right)=\sum_{\tau }G\left(\pi ,\tau \right)\\ G\left(\pi ,\tau \right)=E_{\tilde{Q}}\left[\text{ln }Q\left(s_{\tau }|\pi \right)-\text{ln }Q\left(s_{\tau }|o_{\tau },\pi \right)-\text{ln }P\left(o_{\tau }\right)\right]\\ =\underset{\left(negative\right)\;mutual\;information}{\underset{︸}{E_{\tilde{Q}}\left[\text{ln }Q\left(s_{\tau }|\pi \right)-\text{ln }Q\left(s_{\tau }|o_{\tau },\pi \right)\right]}}-\underset{expected\;log\;evidence}{\underset{︸}{E_{\tilde{Q}}\left[\text{ln }P\left(o_{\tau }\right)\right]}}\\ =\underset{\left(negative\right)\;epistemic\;value}{\underset{︸}{E_{\tilde{Q}}\left[\text{ln }Q\left(o_{\tau }|\pi \right)-\text{ln }Q\left(o_{\tau }|s_{\tau },\pi \right)\right]}}-\underset{extrinsic\;value}{\underset{︸}{E_{\tilde{Q}}\left[\text{ln }P\left(o_{\tau }\right)\right]}}\\ =\underset{expected\;cost}{\underset{︸}{D\left[Q\left(o_{\tau }|\pi \right)||P\left(o_{\tau }\right)\right]}}+\underset{expected\;ambiguity}{\underset{︸}{E_{\tilde{Q}}\left[H\left[P\left(o_{\tau }|s_{\tau }\right)\right]\right]}} \end{array}$$

where $\tilde{Q}=Q\left(o_{\tau },s_{\tau }|\pi \right)=P\left(o_{\tau }|s_{\tau }\right)Q\left(s_{\tau }|\pi \right)\approx P\left(o_{\tau },s_{\tau }|õ,\pi \right)$ and *Q*(*o*_τ_|*s*_τ_, π) = *P*(*o*_τ_|*s*_τ_) for τ > *t*.

#### Remarks

The expected relative entropy now becomes mutual information or epistemic value, while the expected log-evidence becomes extrinsic value—if we associate the prior preferences with value or utility. The final equality expression shows how expected free energy can be evaluated relatively easily: it is just the divergence between the predicted and preferred outcomes, plus the ambiguity (i.e., entropy) expected under predicted states.

There are several helpful interpretations of expected free energy that appeal to (and contextualize) established constructs. For example, maximizing epistemic value is equivalent to maximizing (expected) Bayesian surprise (Itti and Baldi, 2009), where Bayesian surprise is the Kullback-Leibler (KL) divergence between posterior and prior beliefs. This can also be interpreted in terms of the principle of maximum mutual information or minimum redundancy (Barlow, 1961; Linsker, 1990; Olshausen and Field, 1996; Laughlin, 2001). This is because epistemic value is the mutual information between hidden states and observations. In other words, it reports the reduction in uncertainty about hidden states afforded by observations. Because the information gain cannot be less than zero, it disappears when the (predictive) posterior ceases to be informed by new observations. Heuristically, this means epistemic behavior will search out observations that resolve uncertainty about the state of the world (e.g., foraging to resolve uncertainty about the hidden location of prey or fixating on informative part of a face). However, when there is no posterior uncertainty—and the agent is confident about the state of the world—there can be no further information gain and epistemic value will be the same for all policies, enabling extrinsic value to dominate. This resolution of uncertainty is closely related to satisfying artificial curiosity (Schmidhuber, 1991; Still and Precup, 2012) and speaks to the value of information (Howard, 1966).

The expected complexity or cost is exactly the same quantity minimized in risk sensitive or KL control (Klyubin et al., 2005; van den Broek et al., 2010), and underpins related (free energy) formulations of bounded rationality based on complexity costs (Braun et al., 2011; Ortega and Braun, 2013). In other words, minimizing expected complexity or cost renders behavior risk sensitive, while maximizing expected accuracy induces ambiguity-sensitive behavior. This completes our description of free energy. We now turn to belief updating that is based on minimizing free energy under the generative model described above.

### Belief updating and a neuronal (process) theory

**Figure 3:**
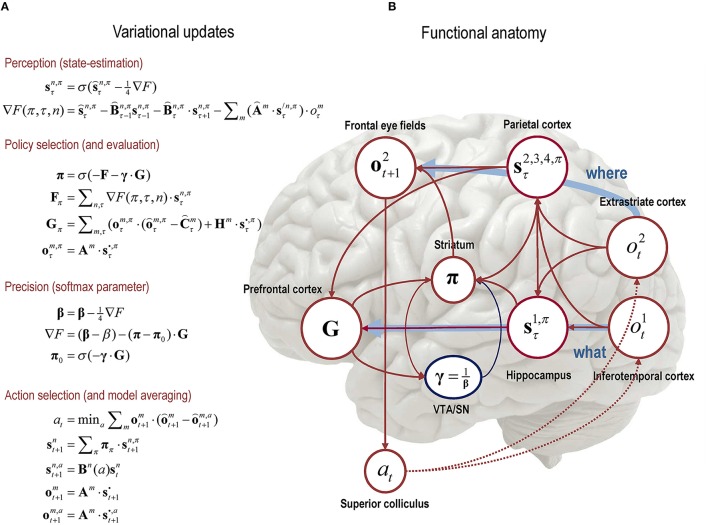
**Schematic overview of the belief updates describing active inference: (A)** The left panel lists the belief updates mediating, perception, policy selection, precision, and action selection; **(B)** while the right panel assigns the quantities that are updated (sufficient statistics or expectations) to various brain areas. The implicit attribution should not be taken too seriously but serves to illustrate the functional anatomy implied by the form of the belief updates. Here, we have assigned observed outcomes to visual representations in the occipital cortex; with exteroceptive (*what*) modalities entering a ventral stream and proprioceptive (*where*) modalities originating a dorsal stream. Hidden states encoding context have been associated with the hippocampal formation, while the remaining states encoding sampling location and spatial invariance have been assigned to the parietal cortex. The evaluation of policies, in terms of their (expected) free energy, has been placed in the ventral prefrontal cortex. Expectations about policies *per se* and the precision of these beliefs have been associated with striatal and ventral tegmental areas to indicate a putative role for dopamine in encoding precision. Finally, beliefs about policies are used to create Bayesian model averages of future outcomes (in the frontal eye fields)—that are fulfilled by action, via the deep layers of the superior colliculus. The arrows denote message passing among the sufficient statistics of each factor or marginal. Please see the text and Table 1 for an explanation of the equations and variables. In this paper, the hat notation denotes a natural logarithm; i.e., $\overset{⌢}{\text{o}}=\text{ln }$o.

In practice, expectations about hidden variables can be updated using a standard gradient descent on variational free energy. Figure 3 provides an example of these updates. It is easy to see that the updates minimize variational free energy because they converge when the free energy gradients are zero: i.e., ∇*F* = 0. Although the updates look complicated, they are remarkably plausible in terms of neurobiological schemes—as discussed elsewhere (Friston et al., 2014). For example, expectations about hidden states are a softmax function (c.f., neuronal activation function) of two terms. The first is a decay term, because the log of a probability is always zero or less ${\overset{⌢}{s}}_{\tau }^{n,\pi }=\text{ln }s_{\tau }^{n,\pi }\leq 0$. The second is the free energy gradient, which is just a linear mixture of (spiking) activity from other representations (expectations). Similarly, the precision updates are a softmax function of free energy and its expected value in the future, weighted by precision or inverse temperature. The expected precision is driven by the difference in expected free energy with and without observations; much like dopamine is driven by the difference in expected and observed rewards (Schultz et al., 1997). See Friston et al. (2015) for further discussion.

The key thing about these updates is that they provide a process theory that implements the normative theory offered by active inference. In other words, they constitute specific processes that make predictions about neuronal dynamics and responses. Although the focus of this paper is on behavior and large-scale functional anatomy, we will illustrate the simulated neuronal responses associated with active inference in later sections.

### Functional segregation and the mean field approximation

An important aspect of the belief updating in Figure 3 is that it is formulated for a particular form of posterior density. This form rests upon something called a *mean field approximation*, which is a ubiquitous device in Bayesian statistics (and statistical physics) (Jaakkola and Jordan, 1998). Figure 1 (right panel) expresses the posterior as a product of independent marginal distributions over different sorts of hidden states (i.e., factors) at different time points. This mean field assumption is quite important: it means hidden states are represented in a compact and parsimonious fashion. In other words, instead of encoding expectations of a full joint distribution over several factors (e.g., where an object is and what an object is), we just need to represent both attributes in terms of their marginal distributions. Similarly, instead of representing the entire trajectory of hidden states over time, we can approximate the trajectory by encoding expectations at each time point separately. This leads to an enormous simplification of the numerics and belief updating. However, there is a price to pay: because the posterior beliefs are conditionally independent, dependencies among the factors are ignored. Generally, this leads to overconfidence, when inferring hidden states—we will see an example of this below.

From a neurobiological perspective, the mean field approximation corresponds to the principle of *functional segregation*, in which representations are anatomically segregated in the cortical mantle (Zeki and Shipp, 1988). A nice example of this is the segregation of ventral and dorsal visual processing streams that deal with “*what*” and “*where*” attributes of a visual scene, respectively (Ungerleider and Mishkin, 1982). In the absence of a mean field approximation, there would be neuronal representations of every possible object in every location. It is this aspect of approximate (variational) Bayesian inference we emphasize in this paper, by sketching the implications for large-scale functional anatomy. The segregation or factorization into “*what*” and “*where*” attributes is particularly prescient for the oculomotor control of saccadic eye movements. This is because—as we will see next—action is only specified by the states or attributes of the world that it can change. Clearly, saccadic eye movements only change where one is looking but not what is sampled. This means that only one factor or posterior marginal is sufficient to prescribe action.

For illustrative purposes, Figure 3 shows how the variables in our scheme could be encoded in the brain. The encoding of object identity is assigned to inferotemporal cortex (Seeck et al., 1995). The representation of location is associated with (dorsal) extrastriate cortex (Haxby et al., 1994). Beliefs about sampling locations and spatial invariances are attributed to parietal cortex, which anticipates the retinal location of stimuli in the future and updates the locations of stimuli sampled in the past (Duhamel et al., 1992). Inference about scene identity (based on the spatial relationships among objects) is attributed to the hippocampus (Rudy, 2009). Beliefs about policies are assigned to the striatum (Frank, 2011), which receives inputs from prefrontal cortex, ventral tegmental area and hippocampus to coordinate planning (in prefrontal cortex, Tanji and Hoshi, 2001) and execution (VTA/SN), given a particular context. In active inference, action selection depends upon the precision of beliefs about policies (future behavior), encoded by dopaminergic projections from VTA/SN to the striatum (Schwartenbeck et al., 2014). Frontal eye fields are involved in saccade planning (Srihasam et al., 2009) and the superior colliculus mediates eye movement control (Grossberg et al., 1997)—by fulfilling expectations about action that are conveyed from frontal eye fields.

### Action and behavior

The equations in Figure 3 conclude with a specification of action, where action is selected to minimize the difference (KL divergence) between the outcome predicted under each action—based on beliefs about the current state—and the outcome predicted for the next state. This specification of action is considered reflexive by analogy to motor reflexes that minimize the discrepancy between proprioceptive signals (primary afferents) and descending motor commands or predictions. If we regard competing policies as models of behavior, the expected outcome is formally equivalent to *Bayesian model average* of outcomes, under posterior beliefs about policies.

### Summary

By assuming a generic (Markovian) form for the generative model, it is fairly simple to derive Bayesian updates that clarify the interrelationships between perception, policy selection, precision and action. In brief, the agent first infers the hidden states under each model or policy that it entertains. It then evaluates the evidence for each policy based upon prior beliefs or preferences about future states. Having optimized the confidence in beliefs about policies, their expectations are used to form a Bayesian model average of the next outcome, which is realized through action. The anatomy of the implicit message passing is not inconsistent with functional anatomy in the brain: see Friston et al. (2014) and Figure 3. Figure 3 shows the functional anatomy implied by the belief updating and mean field approximation in Figure 1. Here, we have assumed two input modalities (*what* and *where*) and four sets of hidden states; one encoding the content of the visual scene and the remaining three encoding the location at which it was sampled (and various spatial transformations). The anatomical designation in Figure 3 should not be taken too seriously—the purpose of this illustration is to highlight the recurrent message passing among the expectations that constitute beliefs about segregated or factorized states of the world. Here, we emphasize the segregation between what and where streams—and how the dorsal *where* stream supplies predicted outcomes (to frontal eye fields) that action can realize (via the superior colliculus). The precision of beliefs about policies has been assigned to dopaminergic projections to the striatum. We will use this particular architecture in the next section to illustrate the behavioral (and electrophysiological) responses that emerge under this scheme.

Although the generative model—specified by the (**A,B,C,D**) matrices—changes from application to application, the belief updates in Figure 3 are generic and can be implemented using standard software routines (here, **spm_MDP_VB_X.m**). These routines are available as Matlab code in the SPM academic software: http://www.fil.ion.ucl.ac.uk/spm/. In fact, the following simulations can be reproduced (and modified) by downloading the DEM Toolbox and invoking **DEM_demo_MDP_search.m**. This annotated code can also be edited and executed via a graphical user interface; by typing >> DEM and selecting the **Visual foraging** demo. This demo can be compared with the equivalent variational filtering scheme (for continuous state-space models) in the **Visual search** demo, described in Friston et al. (2012).

## Active inference and visual foraging

This section uses active inference for Markov decision processes to illustrate epistemic foraging in the setting of visual searches. Here, the agent has to categorize a scene on the basis of the relative position of various visual objects—that are initially unknown. Crucially, the agent can only sample one object or location at a time and therefore has to accumulate evidence for competing hypotheses about the underlying scene. When the agent is sufficiently confident about its perceptual categorization, it makes a saccade to a choice location—to obtain feedback (“*right*” or “*wrong*”). *A priori*, the agent prefers to be right and does not expect to be wrong. We first illustrate a single trial in terms of behavior and underlying electrophysiological responses. The next section then considers sequences of trials and how average behavior (accuracy, number of saccades and saccadic intervals) depends upon prior preferences and precision.

This demonstration uses a mean field approximation to the posterior over different hidden states (*context, sampling location*, and *spatial transformations*). In addition, we consider two outcome modalities (exteroceptive or “*what*” and proprioceptive or “*where*”). In this example, the agent has to categorize a scene that comprises cues at four peripheral locations, starting from a central fixation point. This involves a form of scene construction, in which the relationship between various cues determines the category of scene. The scene always contains a bird and seed, or a bird and a cat. If the bird is next to the seed or the cat, then the scene is categorized as “*feed*” or “*flee*,” respectively. Conversely, if the seed is diagonally opposite the bird, the category is “*wait*.” The particular positions of the cues are irrelevant; the important attributes are their spatial relationship. This means hidden states have to include spatial mappings that induce invariances to spatial transformations. These are reflections around the horizontal and vertical axes.

The right panel of Figure 2 shows the hidden states in more detail: there are two outcome modalities (*what* and *where*), encoding one of six cues (*distractor, seed, bird, cat*, and *right* or *wrong* feedback) and one of eight sampled locations (central *fixation*, four quadrants, and three *choice locations* that provide feedback about the respective decision). The hidden states have four factors; corresponding to context (*feed, flee*, and *wait*), the currently sampled location (the eight locations above) and two further factors modeling invariance (i.e., with and without reflections about the vertical and horizontal axes). The three scenes under each context (flee, feed and wait) in the top right panel of Figure 2 are referred to as base scenes. The context or category defines the objects (distractor, seed, bird, and cat) and their relative locations. The hidden states mediating (vertical and horizontal) transformations define the absolute locations and are implemented with respect to the base scenes. For example, in the case of a flee scene, the bird and cat may exchange locations under a vertical transformation. Since the absolute and relative positions of the objects (and the objects themselves) are hidden causes of the scene's appearance, they are not affected by the agent's actions.

Heuristically, the model in Figure 2 generates outcomes in the following way. First, one of the three canonical scenes or contexts is selected. This scene can be flipped vertically or horizontally (or both) depending upon the spatial transformation states. Finally, the sampled location specifies the exteroceptive visual cue and the proprioceptive outcome signaling the location. This model can be specified in a straightforward way by specifying the two outcomes for every combination of hidden states in **A**^1^ ∈ ℝ^6×(3×8×2×2)^ and **A**^2^ ∈ ℝ^8×(3×8×2×2)^. The arrays in these two matrices just contain a one for each outcome when the combination of hidden states generates the appropriate outcome, and zeros elsewhere. These two matrices encode the observation likelihoods in the two outcome modalities *what* and *where*. Here, **A**^1^ defines the identity (*what*) of objects that are likely to be sampled (i.e., observed), under all possible combinations of hidden states, while **A**^2^ defines the likely locations (*where*) of the objects. The transition matrices are similarly simple: because the only state that changes is the sample location, the transition matrices are identity matrices, apart from the (action dependent) matrix encoding transitions among sampled locations:

$$B_{ij}^{2}(k)={\mathbb{R}}^{(8\times 8)\times 8}=\{\begin{array}{cc} 1, & i=k\\ 0, & i\neq k \end{array}$$

where *k* ∈ {1, 2, …, 8}. Prior beliefs about the initial states **D**^*n*^ (context and projections) were uniform distributions; apart from the sampled location, which always started at the central fixation **D**^2^ = [1, 0, …, 0]. Here, n indicates the dimension of the hidden states with *n* ∈ {1, 2, 3, 4}. There are four dimensions of hidden states, namely context, sampling location, and the two spatial transformations.

Although we have chosen to illustrate a particular paradigm, the computational architecture of the scheme is fairly generic. Furthermore, after the generative model has been specified, all its parameters are specified through minimization of free energy. This means there are only two parameters that can be adjusted; namely, prior preferences about outcomes, C and prior precision, γ. In our case, the agent has no prior preference (i.e., flat priors) about locations but believes that it will correctly categorize a scene after it has accumulated sufficient evidence. Prior preferences over the outcome modalities were therefore used to encourage the agent to choose policies that elicited correct feedback *C*^1^ = [0, …, 0, *c*, −2*c*]:*c* = 2, with no preference for sampled locations *C*^2^ = [0, …, 0]. Here, *c* is the utility of making a correct categorization, −2*c* is the utility of being wrong. These preferences mean that the agent expects to obtain correct feedback exp(*c*) times more than visual cues—and believes it will solicit incorrect feedback very rarely. The prior precision of beliefs about behaviors (policies or future actions) γ plays the role of an inverse temperature parameter. As the precision increases, the sampling of the next action tends to be more deterministic; favoring the policy with the lowest expected free energy. Conversely as the precision of beliefs decreases the distribution of beliefs over the policies becomes more uniform; i.e., the agent becomes more uncertain about the policy it is pursuing.

With these preferences, the agent should act to maximize epistemic value or resolve uncertainty about the unknown context (the scene and its spatial transformations), until the uncertainty about the scene is reduced to a minimum. At this point, it should maximize extrinsic value by sampling the choice location it believes will provide feedback that endorses its beliefs. This speaks to the trade-off between exploration and exploitation. Essentially, actions associated with exploration of the scene (one of four quadrants) have no extrinsic value—they are purely epistemic. In contrast, actions associated with the choice locations (locations that are used to report the scene's category) have extrinsic value, because the agent has prior preferences about the consequences of these actions. The contributions of epistemic and extrinsic value to policy (and subsequent action) selection are determined by their contributions to expected free energy (see Equation 2). In other words, there is only one imperative (to minimize free energy); however, free energy can always be expressed as a mixture of epistemic and extrinsic value. The relative contribution is determined by the precision of prior preferences, in relation to the epistemic part. The exploration and exploitation dilemma is resolved such that when the extrinsic value of the policies associated with a choice is greater than the epistemic value, the agent terminates the exploration and exploits one of the choice locations (i.e., declares its decision). This reflects a general behavioral pattern during active inference; namely, uncertainty is resolved via minimizing a free energy that is initially dominated by epistemic value—until extrinsic value or prior preferences dominate and exploitation supervenes. Notice that pragmatic behavior (choice behavior) is driven by preferences in one modality (exteroceptive outcomes), while action is driven by predictions in another (proprioceptive sampling location). Despite this, action brings about preferred outcomes. This rests upon the recurrent belief updating that links the “*what*” and “*where*” streams in Figure 3. In this graphic, we have assumed that proprioceptive information has been passed from the trigeminal nucleus, via the superior colliculus to visual cortex (Donaldson, 2000).

### Simulating saccadic searches

**Figure 4:**
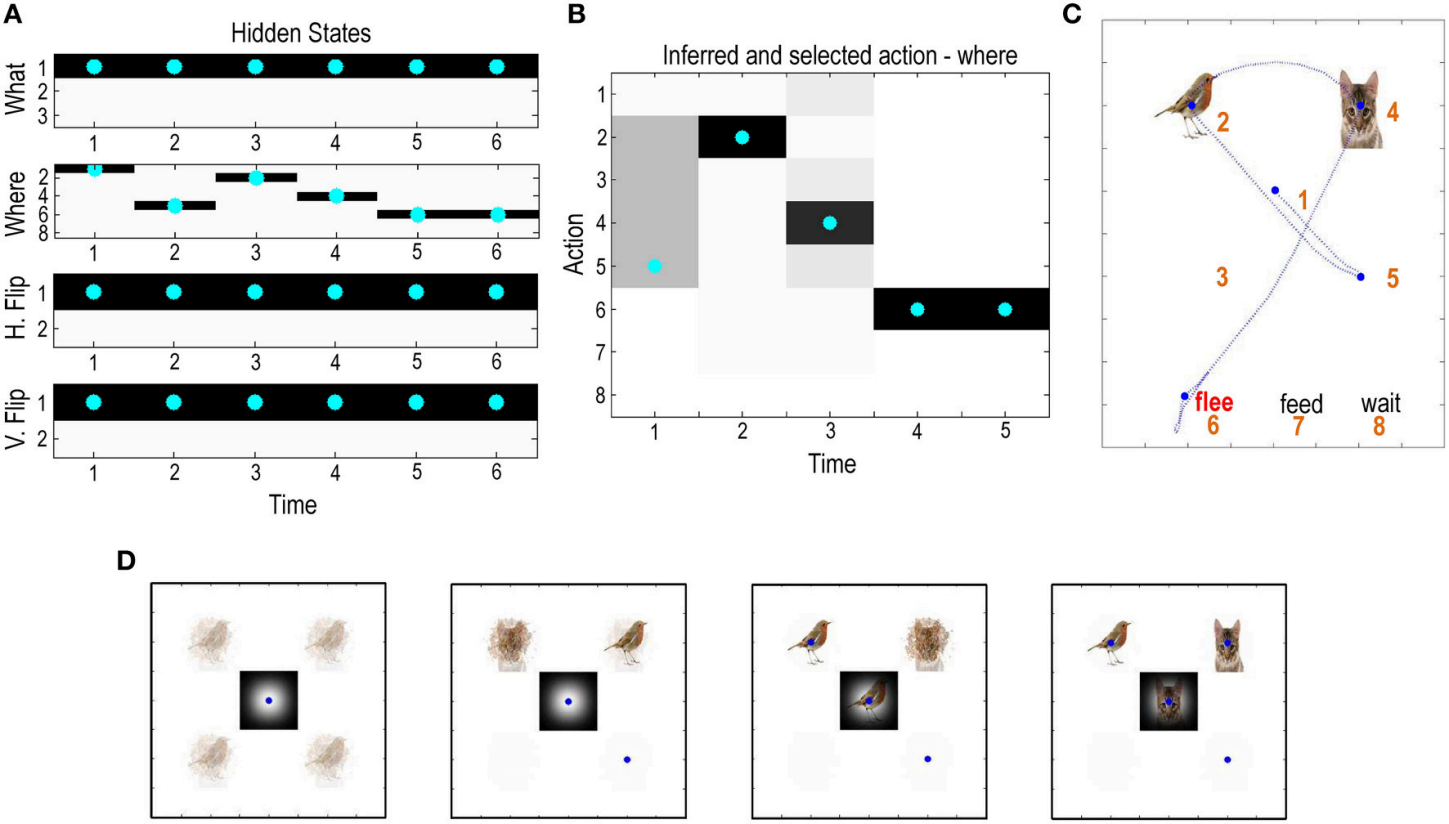
**Simulated visual search: (A)** This panel shows the expectations about hidden states and the expectations of actions are shown in **(B)** (upper middle), producing the search trajectory in **(C)**—after completion of the last saccadic movement. Expectations are shown in image format with black representing 100% probability. For the hidden states each of the four factors or marginals are shown separately, with the true states indicated by cyan dots. Here, there are five saccades and the agent represents hidden states generating six outcomes (the initial state and five subsequent outcomes). The results are shown after completion of the last saccadic, which means that, retrospectively, the agent believes it started in a *flee* context, with no horizontal or vertical reflection. The sequence of sampling locations indicates that the agent first interrogated the lower right quadrant and then emitted saccades to the upper locations to correctly infer the scene—and make the correct choice (indicated by the red label). The lower panel **(D)** illustrates the beliefs about context during the first four saccades. Initially, the agent is very uncertain about the constituents of each peripheral location; however, this uncertainty is progressively resolved through epistemic foraging, based upon the cues that are elicited by saccades (shown in the central location). The blue dots indicate the sampling location after each saccade.

Figure 4 shows the results of updating the equations in Figure 3, using 16 belief updates between each of five saccades. Beliefs about hidden states are updated using a gradient descent on variational free energy. This gradient descent usually converges to a minimum within about 16 iterations. We therefore fixed the number of iterations to 16 for simplicity. This imposes a temporal scheduling on belief updates and ensures that the majority (here, more than 80%) of epochs attain convergence (this convergence can be seen in later figures, in terms of simulated electrophysiological responses). The belief updates are shown in terms of posterior beliefs about hidden states (upper left panels), posterior beliefs about action (upper center panel) and the ensuing behavior (upper right panel). Here, the agent constructed policies on-the-fly by adding all possible actions (saccadic movement to the eight possible locations) to previous actions. This means that the agent only looks one move ahead—and yet manages to make a correct categorization in the minimum number of saccadic eye movements: in this trial, the agent first looks to the lower right quadrant and finds a distractor (omitted in the figures for clarity). It then samples the upper quadrants to resolve uncertainty about the context, before soliciting feedback by choosing the (correct) choice location. The progressive resolution of uncertainty over the three initial saccades is shown in more detail in the lower panels.

Here, posterior beliefs about the state of the world (the nature of the canonical scene and spatial transformations) are illustrated graphically by weighting the predicted visual cue—under each state—in proportion to the posterior beliefs about that state. Each successive image reports the posterior beliefs after the first three saccades to the peripheral locations, while the insert in the center is the visual outcome after each saccade. Initially, all four peripheral cue locations could contain any of the visual objects; however, after the first saccade to the lower right quadrant, the agent believes that the objects (bird and seed or cat) are in the upper quadrants. It then confirms this belief and resolves uncertainty about vertical reflection by sampling the upper right quadrant to disclose a bird. Finally, to make a definitive decision about the underlying scene, it has to sample the juxtaposed location to resolve its uncertainty about whether this contains seed or cat. Having observed cat, it can then make the correct choice and fulfill its prior beliefs or preferences.

This particular example is interesting because it illustrates the overconfidence associated with a mean field approximation. Note that after the first saccade the agent assumes that the scene must be either a *feed* or *flee* category, with no horizontal reflection. This is reflected in the fact that the lower quadrants are perceived as empty. If the agent was performing exact Bayesian inference it would allow for the possibility of a *wait* scenario, with the bird and seed on the diagonal locations. In this instance, it would know that there must have been either a vertical or horizontal reflection (but not both). However, this knowledge (belief) cannot be entertained under the mean field approximation, because inferring a vertical or horizontal reflection depends on whether or not the scene is a *wait* category. It is these conditional dependencies that are precluded by the mean field approximation; in other words, posterior beliefs about one dimension of hidden states (e.g., reflection) cannot depend upon posterior beliefs about another (e.g., scene category). The agent therefore finds the most plausible explanation for the current sensory evidence, in the absence of conditional dependencies; namely, there has been no vertical reflection and the scene is not “*wait*.” If the brain does indeed use mean field approximations—as suggested by the overwhelming evidence for functional segregation—one might anticipate similar perceptual synthesis and saccadic eye movements in human subjects. In principle, one could compare predictions of empirical eye movements under active inference schemes with and without mean field approximations—and test the hypothesis that the brain uses marginal representations of the sort assumed here (see Discussion).

### Electrophysiological correlates of variational belief updating

**Figure 5:**
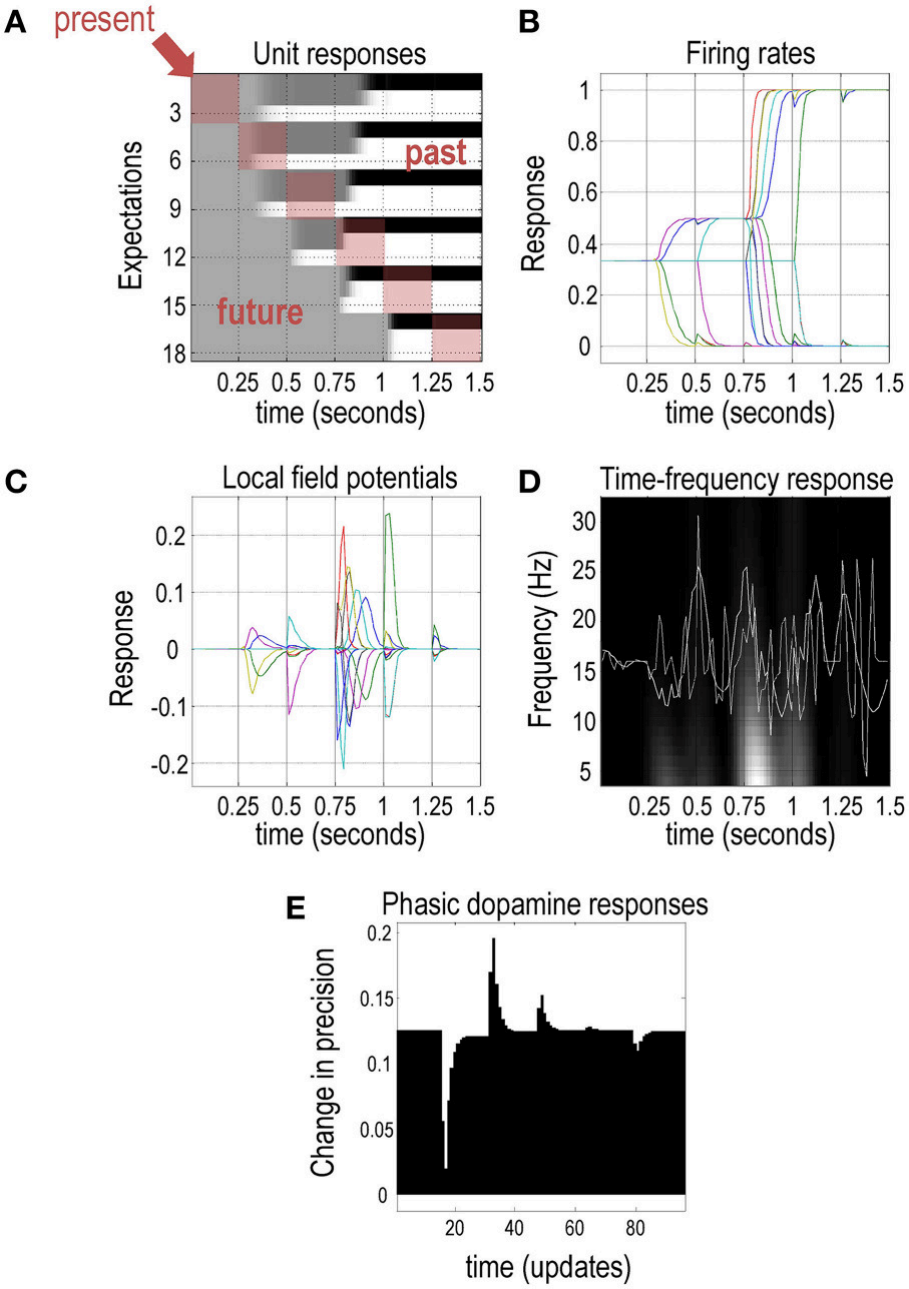
**Simulated electrophysiological responses:** this figure reports the belief updating behind the behavior shown in the previous figure. **(A)** The upper left panel shows the activity (firing rate) of units encoding the context or scene in image (raster) format, over the six intervals between saccades. These responses are organized such that the upper rows encode the probability of alternative states in the first epoch, with subsequent epochs in lower rows. **(B)** The upper right panel plots the same information to illustrate the evidence accumulation and the resulting disambiguation of context. **(C)** The simulated local field potentials for these units (i.e., the rate of change of neuronal firing) are shown in the middle left panel. **(D)** The middle right panel shows average local field potential over all units before (dotted line) and after (solid line) bandpass filtering at 4 Hz, superimposed upon its time frequency decomposition. **(E)** The lower panel illustrates simulated dopamine responses in terms of a mixture of precision and its rate of change.

Figure 5 shows the belief updating during the above visual search to emulate electrophysiological responses measured in empirical studies. The upper left panel shows simulated neuronal activity (firing rates) for units encoding the first (scene category) hidden state using an image (or raster) format. Units here correspond to the expectations (posterior probabilities) about hidden states of the world. There are six hidden states for each of the three scenes (*flee, feed*, or *wait*) at six different times. Crucially, there are two sorts of time shown in these responses. Each block of the raster encodes the activity over 16 time bins (belief updates) between one saccade and the next, with one hidden state in each row. Each row of blocks reports expectations about one of the three hidden states at different times in the future (or past)—here, beliefs about the context following each of the six saccades. Each column of blocks shows the expectations (about the past and future) at a particular point during the trial. This effectively shows the beliefs about the hidden states in the past and the future. For example, the second row of blocks summarizes belief updates about the second epoch over subsequent saccades; i.e., expectations about the context in the second saccade are updated in the following saccades (blocks to the right), while the first column of blocks encodes beliefs about future states prior to emission of the first saccade; i.e., expectations about the context in the second time step is projected into the past (one block above) and into the future (one block below). This means beliefs about the current state occupy blocks along the leading diagonal (highlighted in red), while expectations about states in the past and future are above and below the diagonal, respectively. For example, the color density in the first row denotes the posterior probability of the context being “flee” during the first saccade: this expectation about context prior to the first saccade only becomes definitive at around 0.9 s (during the fourth saccade). Conversely, row 12 denotes the posterior probability of ‘wait’ during the fourth saccade: note that this converges to zero before the fourth saccade has occurred.

This format illustrates the encoding of states over time, emphasizing the implicit representation of the past and future. To interpret these responses in relation to empirical results, one can assume that outcomes are sampled every 250 ms (Srihasam et al., 2009). Note the changes in activity after each new outcome is observed. For example, the two units encoding the first two hidden states start off with uniform expectations over the three scenes that switches after the second and fourth saccade to eventually encode the expectation that the first (*flee*) scene is being sampled. Crucially, by the end of the visual search, these expectations pertain to the past; namely, the context at the start of the trial. In other words, these memories are based upon postdiction. Although not illustrated here, this can be very useful when updating beliefs between trials (when the context does not change).

The upper right panel plots the same information (expectations about the hidden states) to highlight saltatory evidence accumulation, in which expectations diverge as the search progresses. This belief updating is formally identical to evidence accumulation described by drift diffusion or race-to-bound models (de Lafuente et al., 2015; Kira et al., 2015). Furthermore, the separation of timescales implicit in variational updating reproduces the stepping dynamics seen in parietal responses during decision-making (Latimer et al., 2015). The middle left panel shows the associated local field potentials, which are simply the rate of change of neuronal firing shown on the upper right panel. The middle right panel of Figure 5 shows the simulated local field potential averaged over all units before (dotted line) and after (solid line) bandpass filtering at 4 Hz. These responses are superimposed on its time frequency decomposition. The key observation here is that depolarization in the theta range coincides with induced responses—a theme that we pursue elsewhere in terms of theta-gamma coupling in the brain (Canolty et al., 2006; Lisman and Redish, 2009; Friston et al., 2014).

The lower panel illustrates simulated dopamine responses in terms of a mixture of expected precision γ (or equivalently inverse temperature 1∕β) and its rate of change. Here, we see a phasic suppression when the null or distractor cue is sampled after the first saccade, followed by a phasic burst when the bird is seen—and a degree of uncertainty about policies is resolved. A second burst occurs on the third saccade, when the agent resolves uncertainty about the underlying scene (and the decision it will report). Collectively, these simulated electrophysiological responses are not dissimilar to the sorts of responses recorded in empirical studies; however, in this paper, we are primarily interested in modeling (epistemic) behavior. Figure 5 shows some of the expectations that are updated using the scheme presented in the left panel of Figure 3. These simulated electrophysiological responses can be associated with activity in the various brain regions in Figure 2; i.e., expectations about hidden states encoding context $s_{\tau }^{1,\pi }$ with the hippocampus and the expected precision of beliefs γ with the VTA/SN. In the final section, we consider multiple trials and how performance depends upon prior preferences and precision.

## The effects of prior beliefs on performance

**Figure 6:**
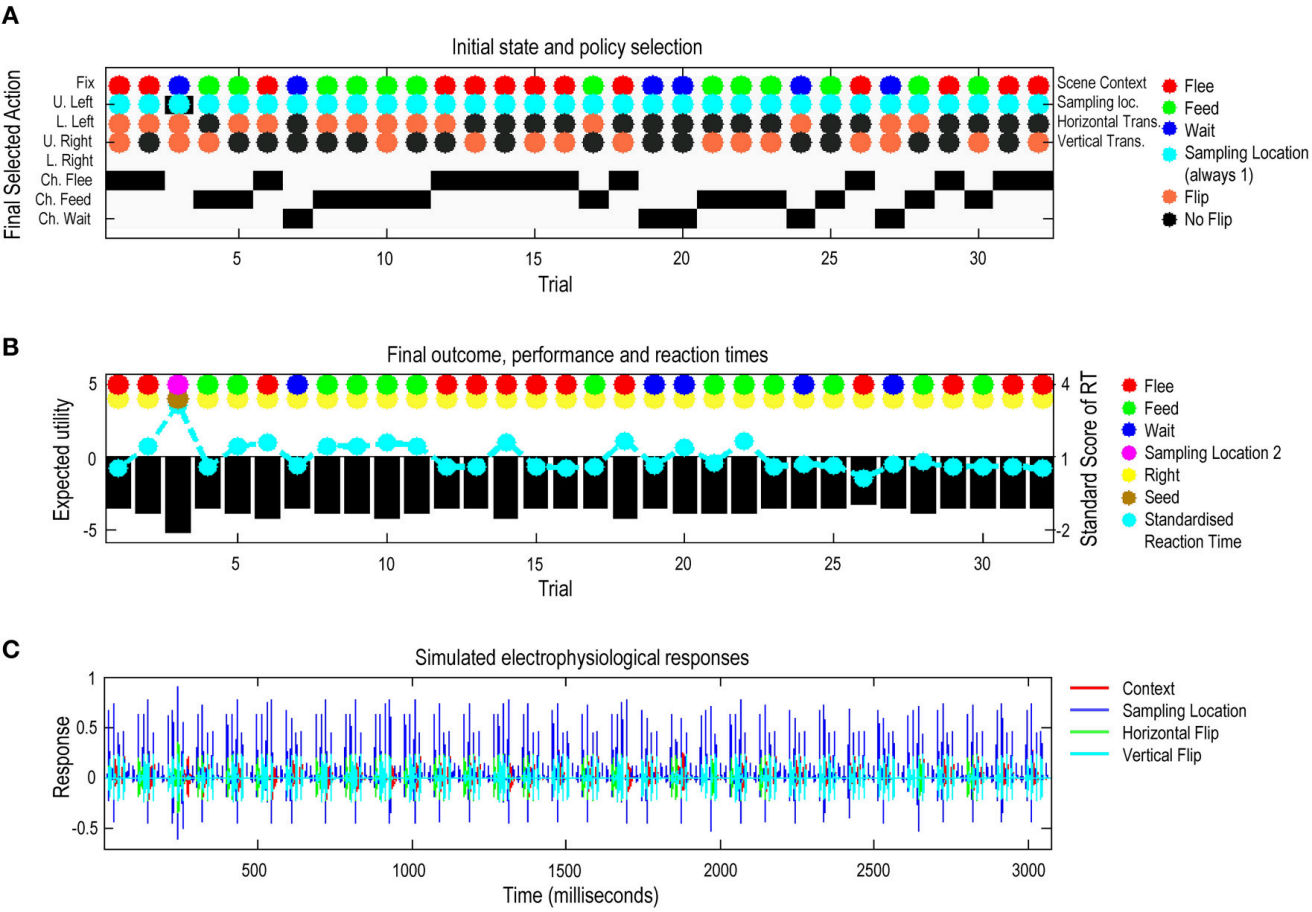
**Simulated responses over 32 trials:** this figure reports the behavioral and (simulated) physiological responses during 32 successive trials. The scenes in these 32 trials were specified via randomly selected hidden states of the world. **(A)** The first panel shows the hidden states of the scene (as colored circles) and the selected action (i.e., the sampled location) on the last saccade. The y-axis on this panel shows two quantities. The selected action is shown using black bars. The agent can saccade to locations one to eight, where the locations six to eight correspond to the choice locations the agent uses to report the scene category. The true hidden states are shown with colored circles. These specify the objects in the scene and their locations (in terms of the context and spatial transformations). The second row of cyan dots indicates that the agent always starts exploring a scene from the central fixation point. Individual rows in the y-axis indicate the sampled locations according to the following: Fix, Fixation; U. Left, Upper left; L. Left, Lower Left; U. Right, Upper Right; L. Right, Lower Right; and Ch. Flee, Choose Flee; Ch. Feed, Choose Feed; and Ch. Wait, Choose Wait. **(B)** The second panel reports the final outcomes (encoded by colored circles) and performance measures in terms of preferred outcomes (utility of observed outcomes), summed over time (black bars) and standardized reaction times (cyan dots). The final outcomes are shown for the sample location (upper row of dots) and outcome (lower row of dots): yellow means the agent made a right choice. **(C)** The third panel shows a succession of simulated event related potentials following each outcome. These are taken to be the rate of change of neuronal activity, encoding the expected probability of hidden states encoding context (i.e., simulated hippocampal activity).

Figure 6 summarizes the (simulated) behavioral and physiological responses over 32 successive trials in which the context (scene and spatial transformations) was selected at random. Each trial comprises six saccades following an initial fixation. The first panel shows the initial states on each trial as colored circles for each of the four marginal hidden states: context, sampling location (always central fixation first), horizontal, and vertical flips and subsequent policy selection (in image format) over the eight actions (i.e., locations) considered at each saccade. Here, the actions one to five correspond to visiting the central fixation point and quadrants with cues (locations two to five), whereas, actions six to eight select the locations reporting the choice (flee, feed, and wait). Choice locations are just there to enable the agent to report its beliefs about the scene category. The second panel reports the agent's decision about the category of the scene and whether this categorization is correct (encoded by colored circles) and performance in terms of expected utility and reaction time. Expected utility (black bars) is the utility of the observed outcomes averaged over time. The utility of an outcome is defined by the prior preference. Note, that because preferences are log probabilities they are always negative—and the best outcome is zero. The performance measure differs across trials because the number of saccades the agent employs before categorizing a scene differs from trial to trial. The reaction times or saccadic intervals (cyan dots) here are based upon the actual processing time in the simulations and are shown after normalization to a mean of zero and standard deviation of one. Our definition of reaction time as the actual processing time (using Matlab *tic-toc* facility) in the simulations is based upon the assumption that belief updates in the brain—via neuronal message passing—follow a similar scheduling to the exchange of sufficient statistics described in Figure 3.

These simulations show that, with the exception of the third trial, the agent makes veridical decisions on every occasion. Interestingly, the third (incorrect) trial is associated with the greatest reaction time. Reaction time here varies because the minimization of free energy converges at a certain tolerance (here, the variational updates terminate when the decrease in free energy falls below 1/128). The lower panel shows the simulated electrophysiological responses using the same format as in the previous figure. Here, we see bursts of high-frequency activity every 100 ms or so; in other words, a nesting of gamma activity in the alpha range.

**Figure 7:**
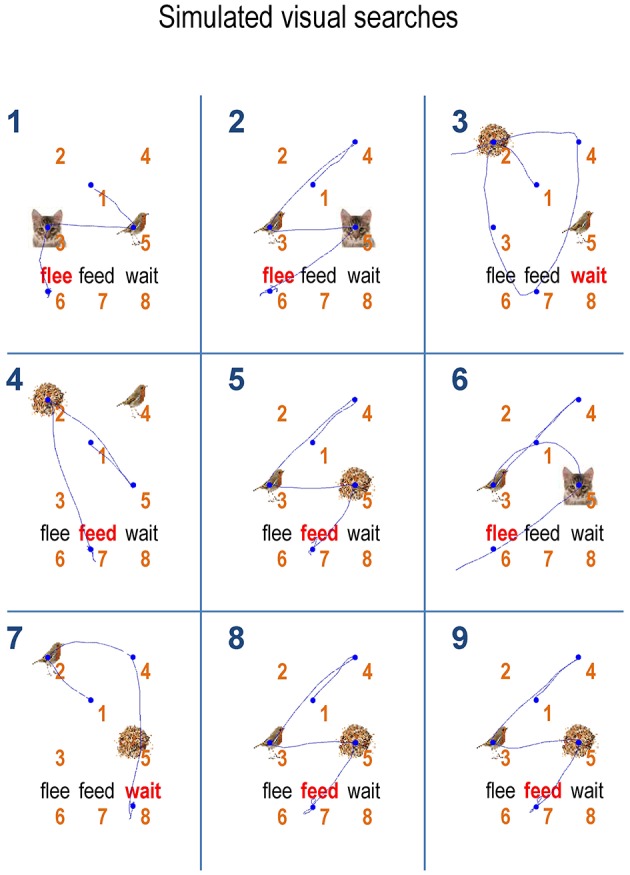
**Sequences of saccades:** this figure illustrates the behavior for the first nine trials shown in the previous figure using the same format as Figure 4 (upper right panel). The numbers on the top left in each cell show the trial number. With the exception of the third trial, the agent is able to recognize or categorize the scene after a small number of epistemically efficient saccades.

The associated behavior, over the first nine trials is depicted in Figure 7. Again, with the exception of the third trial, we see optimal search behavior, with a correct choice after the minimum number of saccades. For example, on the first trial, the first saccade samples a bird, which just requires a second saccade to the adjacent location in order to completely disambiguate the context. A detailed analysis of the belief updating for the failed trial suggested that this was an unlucky failure of the mean field approximation; particularly the factorization over time—and a partial failure of convergence due to the use of a fixed number (i.e., 16) of iterations. These sorts of failures highlight the distinction between exact Bayesian inference and approximate Bayesian inference that may underlie bounded rationality in real agents. With these simulated responses is at hand, we can now assess the effects of changing prior preference and priors over the precision of beliefs about action or policies.

Clearly, there are many model parameters (and hyperparameters) we could consider, in terms of their effects on simulated behavior. We focused on the precision of preferences and policies because these correspond intuitively to the different aspects of salience that may be aberrant in schizophrenia. Motivational salience can be associated with the preferences that incentivise choice behavior. Conversely, the precision of beliefs about policies speaks to the visual salience associated with information gain and epistemic value. Heuristically, one might expect different patterns of behavior depending upon whether subjects have imprecise preferences (i.e., are not confident about what to do), as opposed to imprecise beliefs about the consequences of their actions (i.e., not confident about how to do it). In what follows, we address this heuristic using simulated behavior.

### The effect of priors

**Figure 8:**
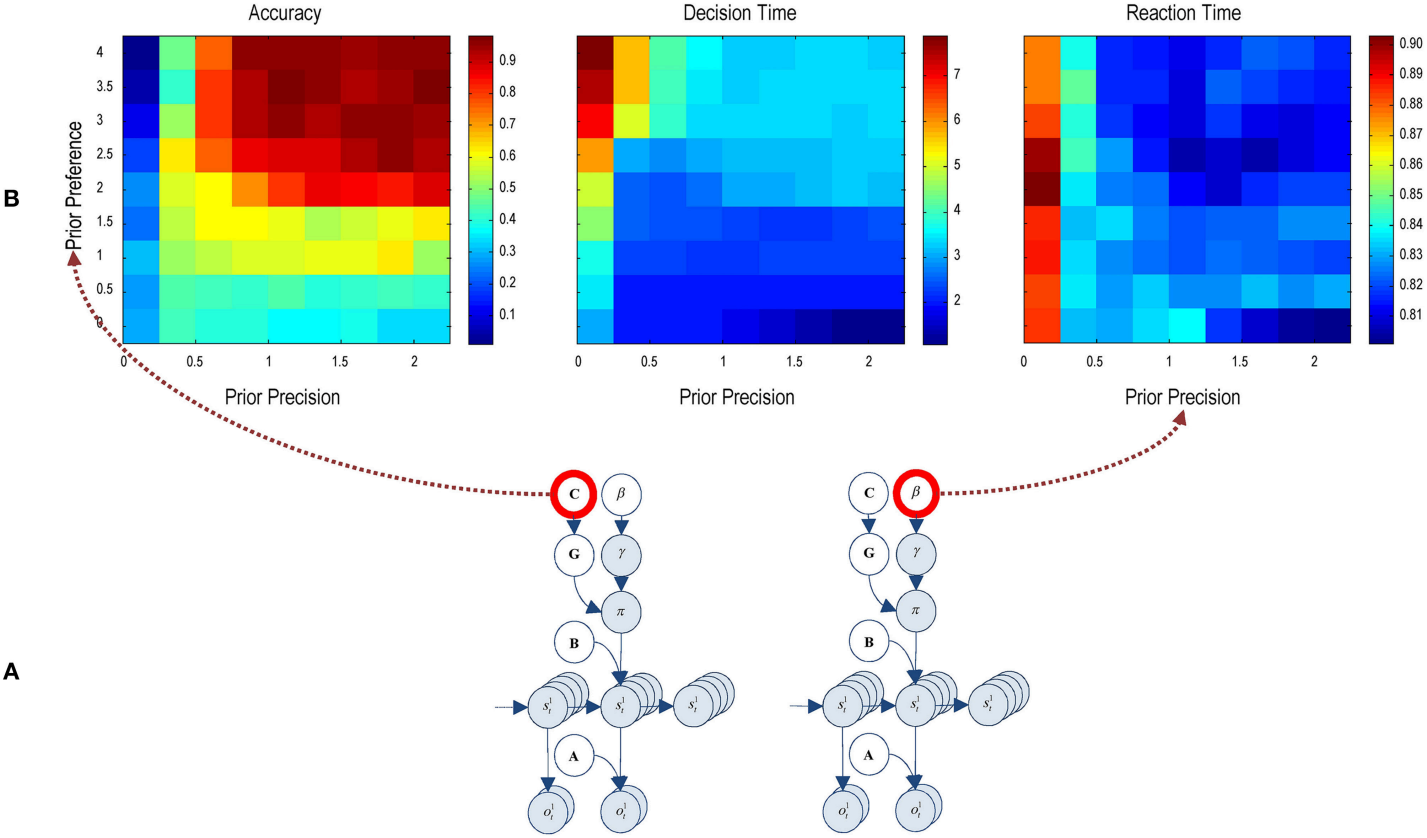
**Performance and priors:** this figure illustrates the average performance over 300 trials. **(A)** The insert (lower panel) shows the prior parameters that were varied; namely, prior preference and precision. These parameters are varied over eight levels. **(B)** For each combination, the accuracy, decision and reaction time were evaluated using simulations (upper row). The accuracy is expressed as the percentage of correct trials (defined as a correct choice in the absence of a proceeding or subsequent incorrect choice). Decision time is defined in terms of the number of saccades until a (correct or incorrect) decision. Reaction time or the interval between saccades is measured in seconds and corresponds to the actual computation time during the simulations.

Finally, Figure 8 reports the performance during presentations of 300 trials, where hidden states of the world were selected randomly—and we allowed the agent to make up to 8 saccades. We measured the performance over these trials in terms of percent accuracy (a correct choice in the absence of an incorrect choice), decision time or number of saccades until any (correct or incorrect) choice and reaction time or saccadic interval (measured in seconds). Here, we repeated the 300 trial paradigm over all combinations of eight levels of prior preference and precision. To manipulate the precision of preferences, we increased the parameter *c*—specifying the prior preferences for different outcomes—from 0 to 4 (i.e., no preferences to very precise preferences).

The left panel in Figure 8 (Accuracy) shows that accurate categorization requires both precise preferences and a high precision. Interestingly, precise prior preferences degrade accuracy when the prior precision is very low. With greater prior preference, the agent does not want to make mistakes. However, a low prior precision precludes a resolution of uncertainty about the scene. The combination of these two priors discourages the agent from making a choice, resulting in an incorrect categorization. The trials where agent doesn't attempt to categorize the scene are considered an incorrect categorization. When prior preferences are less precise, the agent is less afraid of making an incorrect choice, resulting in an improvement in performance but it is still below the chance level. Similarly, greater prior precision does not improve accuracy when prior preference is low. In short, the agent only respond accurately when prior preference and precision are high, as seen on the upper right portion of the image.

The center panel (Decision Time) shows decision time in terms of number of saccades before choosing a choice location. When prior preferences are high and prior precision is very low (first column), it takes seven or eight saccades for the agent to make a decision. Comparing this figure with the accuracy results, it can be seen that accuracy is low even though the agent is making more saccades; i.e., taking its time. When prior precision is high but prior preference is very low, the agent rushes to make a decision—but in the absence of precise prior preferences it makes mistakes (see left panel). In short, the agent successfully categorizes a scene when it deploys three to four saccades (upper right quadrants), under precise preferences and high precision.

The right panel (Reaction Time) shows the reaction time in terms of actual processing time of the simulations. Although, quantitatively, reaction times only vary between about 800 and 900 ms, there seems to be a systematic effect of prior precision, with an increase in reaction time at very low levels.

Crucially, results demonstrate a distinct dependency of accuracy and decision time on prior preference and prior precision. This speaks to the possibility of distinct behavioral phenotypes that are characterized by different combinations of prior preference and precision. For example, agents who do not expect themselves to make mistakes may choose more assiduously, inducing a classical speed accuracy trade-off. Conversely, subjects with more precise beliefs about their choices may behave in a more purposeful and deliberate fashion, taking less time to obtain preferred outcomes. We pursue this theme in the discussion.

## Discussion

In summary, we have presented an active inference formulation of epistemic foraging that provides a framework for understanding the functional anatomy of visual search entailed by sequences of saccadic eye movements. This formulation provides an elementary solution to the problem of scene construction in the context of active sensing and sequential policy optimization, while incidentally furnishing a model of spatial invariance in vision.

Although the problem considered above is relatively simple, it would confound most existing approaches. For example, reinforcement learning and optimal control theories are not applicable because the problem is quintessentially epistemic (belief-based) in nature. This means that the optimal action depends on beliefs or uncertainty about hidden states. This context sensitivity precludes any state-action policy and implicitly any scheme based on the Bellman optimality principle (Bellman, 1952). This is because the optimal action from any state depends upon beliefs about that state and all others. Although, in principle, a belief-state (partially observed) Markov decision process could be entertained (Bonet and Geffner, 2014), the combinatorics of formulating beliefs states over 3 × 8 × 2 × 2 = 96 hidden states are daunting. Furthermore, given the problem calls for sequential policy optimization—and that five moves are necessary to guarantee a correct categorization—one would have to evaluate 8^5^ = 32768 policies.

The active inference solution offered here is based upon minimizing the path-integral of (expected) free energy under a mean field approximation. The exciting thing about this approach is that, computationally, it operates (nearly) in real-time. For example, the reaction times in Figure 8 are based on the actual computation time using a standard desktop personal computer. This computational efficiency may be useful for neurorobotic applications. Having said this, the primary motivation for developing this scheme was to characterize empirical (human) visual searches given observed performance, eye movement, and electrophysiological responses.

The example in this paper has some limitations: for example, all potential spatial combinations of objects can be obtained using just two transformations (e.g., the cat can never be below the bird), and scenes in larger grid worlds may not be describable in terms of simple transformations from a small number of contexts. Clearly, the brain does not use the mean field approximation used to illustrate the scheme—but questions about different forms of meaningful approximations can, in principle, be answered empirically using Bayesian model comparison of such approximations when explaining behavioral or neuroimaging data.

This toy example shows how a scene comprising 2 × 2 quadrants can be explored using the resolution of uncertainty. A scene of this small size could be explored systematically, if inefficiently, (e.g., in a clockwise manner) or by just visiting all locations randomly. However, more complex scenes—which we hope to use in future work—could not be categorized efficiently in such a fashion. In future work, we intend to expand the scene in terms of its size and contents, while retaining the same (active inference) formulation of exploration and ensuing categorization. Our hope is to characterize different behavioral phenotypes, defined in terms of the free parameters of this model; namely, the prior preferences and precision. This paradigm will be used to test the aberrant salience hypothesis of schizophrenia. For that purpose, the experimental design will include task irrelevant distractors (as opposed to the null cues used above), probabilistic relationships between the contents of the scene and its category—and a greater number of cue locations. In principle, this will allow us to explain the difference between normal and schizotypal visual searches in terms of prior preferences, prior precision or a mixture of the two.

Although the accuracy, number of saccades and saccadic intervals (Figure 8) provide a degree of validation for active inference in this setting, it is unlikely that these responses will provide an efficient estimate of subject-specific priors, such as prior preferences and precision. However, it is relatively easy to fit the individual saccadic eye movements by evaluating the probability of *each saccade* in relation to posterior beliefs about action, using the history of action and outcomes in the model above. This means, in principle, it should be possible to estimate things like prior preference and precision efficiently, given the sequence of eye movements from any subject. In subsequent work, we will use the active inference scheme described in this paper to explain empirical eye movements in terms of subject-specific priors. This enables one to simulate or model electrophysiological responses or identify the regional correlates of belief updating, using functional magnetic resonance imaging. This speaks to the ultimate aim of this work, which is to provide a computational phenotyping of individuals, in the hope of characterizing the (formal or computational) psychopathology of conditions like addiction and schizophrenia.

## Author contributions

MM, CM, RA conceived the idea. KF, MM, RA, CM contributed to the writing. KF, MM conducted the simulations.

## Funding

MM, RA, and KF are members of PACE (Perception and Action in Complex Environments), an Innovative Training Network funded by the European Union's Horizon 2020 research and innovation programme under the Marie-Sklodowska-Curie Grant Agreement No 642961. CM is funded by the Max Planck Society. KF is funded by the Wellcome trust (Ref: 088130/Z/09/Z).

### Conflict of interest statement

The authors declare that the research was conducted in the absence of any commercial or financial relationships that could be construed as a potential conflict of interest.
